# Histone and non-histone (de)acetylation impact on the blood-brain barrier

**DOI:** 10.1186/s12987-026-00763-z

**Published:** 2026-01-21

**Authors:** Mariana Melo, Ana Fortuna, João Laranjinha, Joana Bicker

**Affiliations:** 1 Faculty of Pharmacy, University of Coimbra, Coimbra, Portugal; 2 CIBIT - Coimbra Institute for Biomedical Imaging and Translational Research, University of Coimbra, Coimbra, Portugal; 3 Center for Neuroscience and Cell Biology, University of Coimbra, Coimbra, Portugal

**Keywords:** Blood-brain barrier, Epigenetics, Histone acetylases, Histone acetylase activators, Histone deacetylases, Histone deacetylase inhibitors

## Abstract

**Background:**

The blood-brain barrier (BBB) ensures the homeostasis of the central nervous system by regulating the composition of the brain interstitial fluid, required for proper brain function. Nonetheless, its properties are dynamic and susceptible to the influence of environmental factors acting through epigenetic mechanisms, among which are post-translational histone modifications.

**Main body:**

The activity of histone acetylases (HATs) and histone deacetylases (HDACs) on histone and non-histone substrates can alter gene transcription in brain endothelial cells, pericytes, astrocytes and microglia, leading to protective or detrimental effects on BBB integrity and function. These effects may range from the stabilization of intercellular junction proteins in brain endothelial cells to the modulation of neuroinflammation, with consequences on cognitive processes of memory and learning. Ultimately, the positive or negative outcome of HAT/HDAC activity is often context-dependent and varies across different pathologies, according to which corepressors or coactivators are recruited in intracellular signaling cascades, and their subsequent influence on gene expression.

**Conclusions:**

HATs/HDACs modulate the structural integrity and function of the BBB. Further studies in physiologically relevant BBB models are required, in order to provide greater mechanistic insight and overcome translational difficulties.

## Background

**Fig. 1:**
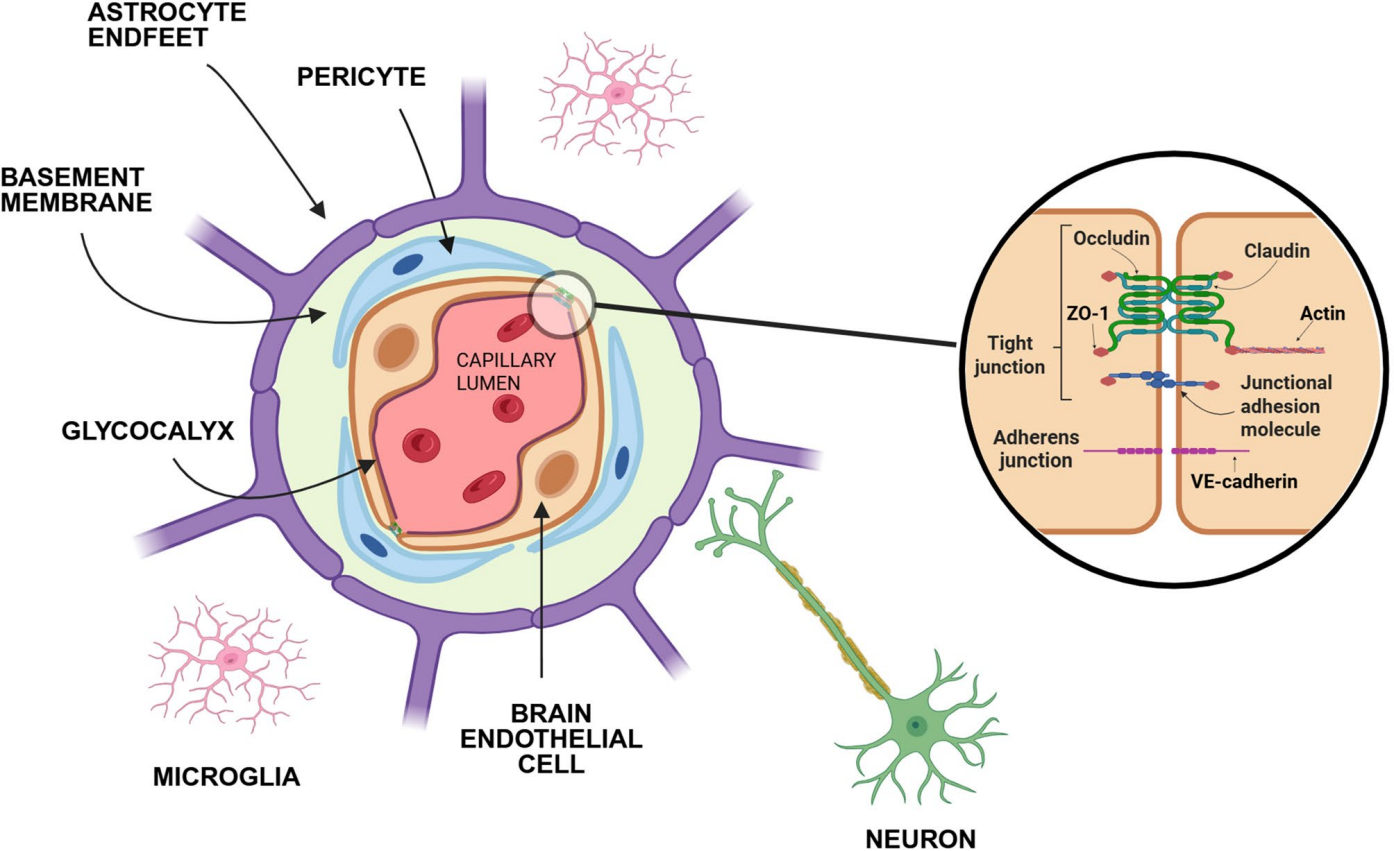
Structural representation of the blood-brain barrier (BBB): schematic illustration of a transverse section showing key components of the BBB and neurons. VE, vascular endothelial; ZO, zonula occludens. Created with BioRender^®^

The brain requires a highly controlled microenvironment to properly perform vitalactivities related to cognition, metabolism regulation, and coordination of central andperipheral functions. These processes rely on a precise communication among neural cells, emphasizing the need for a stable composition of extracellular neural fluid [1]. This is achieved through the barriers of the central nervous system (CNS), among which is the blood-brain barrier (BBB), a dynamic interface separating the brain interstitial fluid from the systemic circulation. The BBB regulates the exchange of ions, molecules, and cells between these two compartments, not only protecting the CNS against external insults, but also participating in signaling networks, and ensuring optimal conditions to support complex neural tasks [2]. To achieve this, specialized brain endothelial cells (BECs) line and seal cerebral blood vessels in physical and functional integration with mural cells (including pericytes and vascular smooth muscle cells), astrocytes, microglia, basement membrane, and glycocalyx, forming collectively with neurons, a multicellular neurovascular unit [3–5] (Fig. 1).

Although the BBB is commonly referred to as a physical barrier, it also encloses transport, metabolic, and immunological barriers [6]. Its properties undergo modifications as the BBB adjusts itself in response to multiple extrinsic (e.g., xenobiotics, pathogenic agents, physical stressors and diet) and intrinsic (e.g., neurotransmitters, hormones and immune mediators) signals that oscillate daily, some with known circadian rhythmicity [1, 7, 8]. The cerebral microvasculature possesses a unique phenotype that differentiates it from the peripheral vascular endothelium. It includes the absence of fenestrae, the high expression of tight junctions and adherens junctions between BECs, a low rate of transcytosis and trafficking of immune cells, and a polarized expression of selective membrane transporters and metabolic enzymes. This phenotype underlies a highly selective movement of substances across the BBB [9–11].

In turn, the abluminal side of BECs is surrounded by mural cells, which collectively referto vascular smooth muscle cells that cover penetrating arterioles, transitional mural cells (i.e., ensheathing pericytes), and capillary pericytes (mesh and thin-strand) that cover microvessels with extended thin processes [12]. Pericytes share an inner vascular basement membrane with BECs and are in direct contact with the endothelium at specific points through peg-and-socket junctions, supporting BBB integrity [2, 12]. Beyond their function as a physical component of the BBB, pericytes are multifunctional cells that contribute to the secretion of extracellular matrix; the regulation of cerebral blood flow (CBF), exerting contractile properties on the capillary diameter; the promotion of processes of angiogenesis and proliferation of BECs, and the phagocytic clearance of cellular debris. Additionally, pericytes, identically to smooth muscle cells in arterioles, are targets for neuron-derived messengers that allow a dynamic modulation of the CBF to meet their metabolic demands, a process known as neurovascular coupling [9, 13–15]. Astrocytes further support neural cell functions in several ways, including the regulation of water homeostasis, ion concentrations, and the neurotransmitter pool [1].

Finally, microglia, have a well-known canonical role in the immune surveillance of the CNS, and also participate in maintaining BBB integrity. Although microglia have traditionally been categorized using simplistic dichotomies such as M1 (proinflammatory and neurotoxic) versus M2 (anti-inflammatory and neuroprotective), or resting versus activated, this nomenclature was considered outdated. It has been emphasized that microglia exist in a spectrum of dynamic states that vary across development, plasticity, aging, and disease contexts. This diversity reflects a multilayered molecular complexity, including transcriptomic, epigenomic, proteomic, and metabolomic profiles, that together define microglial states. These states are shaped by intrinsic factors, such as species, sex, and age, as well as by extrinsic influences, including nutrition, microbiota, and drug exposure [16, 17].

CNS diseases are associated with the dysfunction of blood-CNS barriers, including the BBB. These encompass, but are not limited to, neurodegenerative diseases (e.g., Alzheimer’s disease, Parkinson’s disease, and Huntington’s disease), autoimmune diseases (e.g., multiple sclerosis), traumatic brain injury, hemorrhagic and ischemic stroke, epilepsy, bipolar disorder, and depression [5, 7, 11, 18, 19]. The pathological mechanisms behind BBB dysfunction operate on both cellular and molecular levels, with extrinsic signals often exerting their effects through epigenetic processes [13, 20].

The present review analyzes the mounting scientific evidence regarding the role of histone acetyltransferases (HATs) and histone deacetylases (HDACs) on BBB integrity and function. Study limitations, current epigenetic-based therapies, and future directions related to this topic are also discussed.

## Epigenetic mechanisms of histone acetylation and deacetylation

The completion of human genome sequencing in 2003 laid the foundation forunderstanding how the genetic code is expressed. However, it has become increasingly clear that the genome alone cannot fully explain the complexity of gene expression. This realization stems from the understanding that the genome is a dynamic, responsive entity that undergoes reversible chemical and structural alterations, collectively referred to as epigenetic marks, that influence gene activation or silencing without altering the underlying DNA sequence [21–25]. Epigenetic marks may be heritable through cell division and occur in response to aging and environmental/lifestyle factors (e.g., diet, exercise, sleep, and disease states). These reversible chemical marks are essential for interpreting the genome in a context-specific manner, as they profoundly influence its transcriptional potential [21, 22, 26]. Epigenetic modifications occur in both DNA bases and chromatin-associated proteins, particularly histones, thereby promoting changes in the structure of chromatin. A third mechanism is also led by non-coding RNAs (ncRNAs) divided into short ncRNAs (sncRNAs) with less than 200 nucleotides, including microRNAs (miRNAs), transfer RNAs (tRNAs), piwi-interacting RNAs (piRNAs), and small nucleolar RNAs (snoRNAs); and long ncRNAs (lncRNAs) with more than 200 nucleotides [27, 28]. Epigenetic-like modifications can also occur at messenger RNA (mRNA) level, a field now referred to as epitranscriptomics. Table 1 summarizes epigenetic mechanisms, including chromatin remodeling through DNA and histone chemical modifications, as well as the regulatory role of ncRNAs.

**Table 1:** Major epigenetic mechanisms and their consequences on gene expression

Epigenetic modification	Involved enzymes	Mechanism	Effect on gene expression	References
DNA methylation	DNMT1, DNMT3A, DNMT3B, DNMT3L	Covalent addition of a methyl group on the C5 position of cytosine residues (5mC), often at CpG dinucleotides.	Commonly represses gene expression by preventing the binding of regulatory factors to DNA or by recruiting proteins involved in promoting chromatin compaction into heterochromatin. However, in specific contexts, it may also promote the activation of gene expression.	[21, 23]
DNA Oxidation	TET1, TET2, TET3	Oxidation of 5mC to 5hmC, 5fC or to 5caC. These modifications may serve as epigenetic marks or intermediates in DNA methylation.	5hmC promotes both the activation and repression of gene expression. The relevance of 5fC and 5caC in gene expression remain more speculative and less explored; however, there is increasing evidence regarding the intervention of 5caC in gene repression. In the case of 5fC, there is growing evidence of its role in gene activation.	[153–156]
Histone Acetylation	HATs (e.g., p300, CBP, GCN5 and MYST1/KAT6A)	Addition of acetyl groups from acetyl coenzyme A to the N-terminal lysine residues on histone tails (e.g., H3K9ac and H3K27ac).	Activates gene expression.	[21, 31, 39, 157, 158]
Histone Deacetylation	HDACs	Removal of acetyl groups from the N-terminal lysine residues of histones.	Represses gene expression.	[38, 39]
Histone Methylation	HMTs (e.g., KMTS and PRMTS)	Addition of methyl groups to lysine or arginine residues present on the N-terminal tails of histone proteins (e.g., H3K4me3 and H3K27me3).	Promotes both the activation (e.g., H3K4me3) or the repression (e.g., H3K27me3) of gene expression, depending on the specific residue being methylated and the degree of methylation.	[13, 21, 23, 24, 158, 159]
Histone Demethylation	HDTs (e.g., KMDs)	Removes methylation marks from histone tails.	Can activate or repress gene expression.	[160, 161]
Non-coding RNAs (e.g., siRNA, piRNA, lncRNA)	Variable according to ncRNA	ncRNAs can recruit chromatin-modifying complexes that modify histone marks or DNA methylation.	Can activate or repress gene expression.	[21, 23, 162, 163]

5 C, carbon 5; 5caC, 5-carboxylcytosine; 5fC, 5-formylcytosine; 5hmC, 5-hydroxymethylcytosine; 5mC, 5-methylcytosine; CBP, CREB-binding protein; CpG, cytosine-phosphate-guanine; DNMT, DNA (cytosine-5)- methyltransferase; GCN5, General control of amino acid synthesis 5; H3K27ac, Acetylation of lysine 27 on histone H3; H3K27me3, Trimethylation of lysine 27 on histone H3; H3K4me3, Trimethylation of lysine 4 on histone H3; H3K9ac, Acetylation of lysine 9 on histone H3; HATs, Histone acetyltransferases; HDACs, Histone deacetylases; HDTs, Histone demethylases; HMTs, Histone methyltransferases; KMDs, Lysine-specific demethylases; KMTs, Lysine methyltransferases; lncRNA, Long non-coding RNA; MYST1/KAT6A, Member of MYST family / lysine acetyltransferase 6 A; ncRNA, Non-coding RNA; p300, E1A- associated protein p300; piRNA, PIWI-interacting RNA; PRMTs, Protein arginine methyltransferases; siRNA, Small interfering RNA; TET, Ten-eleven translocation methylcytosine dioxygenase

Altogether, the epigenetic chemical modifications reshape chromatin structure and RNA dynamics, affecting the accessibility of genomic regulatory sequences to transcription factors and other molecular complexes that regulate gene expression, ranging from transcription to splicing and translation [29–31]. In the brain, such modifications are essential for converting transient signals into long-term changes in gene expression, in response to physiological and environmental cues, and for processes that are critical for neurodevelopment, synaptic plasticity, learning, and memory formation [27].

To date, hundreds of epigenetic modifications have been identified, some of which areinterdependent, influencing one another to regulate diverse biological processes and disease states, including neurological disorders [30–35]. Given the complexity of multiple epigenetic modifications across the genome, the challenge lies in elucidating the downstream molecular pathways and their phenotypic consequences. In this regard, methylation and acetylation are the best studied modifications. For instance, in glioblastoma, the most aggressive form of astrocytoma (grade IV), a functional interaction between two epigenetic mechanisms was identified. Aberrant DNA methylation and histone acetylation lead to an upregulation of the neurotrophic gene encoding glial cell line-derived neurotrophic factor (GDNF) [36]. Briefly, DNA hypermethylation at a silencer region prevents the binding of the transcription factor cAMP response element-binding protein (pCREB), redirecting it to an enhancer region of the GDNF gene, where it recruits the HAT, CREB-binding protein (CBP). CBP then acetylates histone H3 at both the enhancer and the transcription start site of the GDNF gene, facilitating the transcriptional activation of GDNF, and promoting tumor cell survival and proliferation [36, 37].

It is well-established that histone conformation affects DNA accessibility for transcription and can be modified reversibly, mainly through changes in the side chains of exposed amino acid residues, located in the N-terminal tail domains outside the core. Modifications of this nature include acetylation, methylation, phosphorylation, citrullination, ubiquitination, SUMOylation, adenosine diphosphate-ribosylation, hydroxylation, and others, that contribute to a diverse and versatile histone code [31, 38]. The acetylation and deacetylation of lysine residues in histone protein tails are among the most well-studied post-translational modifications. These processes are controlled by HATs and HDACs, which respectively add or remove acetyl groups. This alters the electrostatic charge of histones, resulting in structural changes that affect their binding to DNA. Acetylation neutralizes the positive charge on the nitrogen atom of a lysine residue in the histone, converting amines into amides and decreasing its ability to bind to negatively charged phosphate groups of DNA. This reduction in binding results in decreased chromatin compaction, facilitating gene transcription. Conversely, deacetylation leads to a more compacted chromatin structure, which restricts access to the transcription site [38, 39]. These two mechanisms work together to sustain biological processes, some of which are involved in neural functions, including neuronal differentiation, activity-dependent synaptic plasticity, and memory formation. In the context of CNS diseases, acetylation/deacetylation are frequently disrupted, contributing to disease progression [21, 40, 41].

Since acetylation and deacetylation are post-translational modifications of general occurrence in the cell, HATs/HDACs can also act upon non-histone proteins such as transcription factors, chaperones, structural proteins, ion channels, receptors, and enzymes, thereby modulating their function, stability, sub-cellular location, and interactions with other proteins. This has an impact on diverse cellular processes that include cell cycle control, apoptosis, and differentiation, as well as neurobiological functions, encompassing neuronal network development, gamma-aminobutyric acid (GABA)ergic neurotransmission, synaptic plasticity, and synaptogenesis. Furthermore, the disturbance of these mechanisms may have pathophysiological consequences, leading to neuroinflammation, neuronal cell loss, gliosis, and/or mossy fiber sprouting [42, 43].

Lysines are the best-known targets for acetylation and HATs/HDACs are often referred to as lysine acetyltransferases (KATs) and lysine deacetylases (KDACs), respectively [38, 44]. KATs can be divided into three major families: p300/CBP, Gcn5-related N-acetyltransferases, and MYST. In turn, KDACs are divided into four classes: class I (HDAC1, HDAC2, HDAC3, and HDAC8); class IIa (HDAC4, HDAC5, HDAC7, and HDAC9) and IIb (HDAC6 and HDAC10); class III, which are silent information regulators (SIR)2 proteins, also known as sirtuins (SIRT; SIRT1-7) and class IV (HDAC11) [38, 45]. The enzymatic activity of HDACs depends on their cofactor usage: classes I, II, and IV require a zinc ion (Zn²⁺), while class III enzymes (SIRT) rely on nicotinamide adenine dinucleotide (NAD⁺) as a cofactor [46].

Both HAT activators and HDAC inhibitors are under investigation for CNS diseases, although data on HAT activators is yet scarce. Several studies resort to these modulators either to demonstrate the impact of epigenetic modifications on the BBB, or as potential pharmacological tools to ameliorate BBB dysfunction (Table 2).

**Table 2:** Epigenetic modulators with known impact on BBB impairment: summary of main mechanisms

Epigenetic modulator	HAT/HDAC [status]	Mechanism	References
HDAC inhibitors (HDACi)			
AMX0035 [sodium phenylbutyrate (NaPB) and ursodoxicoltaurine]	Pan-HDACi [withdrawn]	• NaPB decreased the expression of iNOS, the release of proinflammatory cytokines and ROS, and NF-κB, in reactive mouse microglia and human astroglia • Increased neurotrophins (BDNF and NT-3) in astrocytes via CREB, improving spatial learning and memory	[164, 165]
Belinostat	Pan-HDACi [approved]	• Decreased astrogliosis and neuroinflammation by suppressing the TLR2-MyD88 signaling pathway in EAE mice • Lowered microglial reactivity, expression of pro-inflammatory cytokines (TNF-α, IL-6, IL-1β and MCP-1 mRNA levels), NO production and iNOS levels in vitro • Inhibited the deacetylation of NF-κB p65, maintaining it in an inactive acetylated state, and reduced HDAC3 expression in microglia. HDAC1, HDAC2 or HDAC6 levels were unchanged	[127]
CKD-506	HDAC6 inhibitor [investigational]	• Promoted BBB integrity by increasing the levels of a tight junction protein (occludin) and decreasing pro-inflammatory cytokines (IFN-γ, IL-12, TNF-α, and IL-1β) • Lessened the clinical symptoms and spinal cord damage of EAE in mice	[166]
Entinostat (MS-275)	Class I HDAC inhibitor [investigational]	• Increased the expression of MITF, a disease-associated microglia regulator. This led to higher amyloid β uptake and to the downregulation of pro-inflammatory cytokine MCP-1, associated with cognitive decline • Rescued claudin-5 expression in the nucleus accumbens of stress-susceptible mice through HDAC1 inhibition, counteracting BBB hyperpermeability and depressive behavior • Worsened BBB damage in an animal model of ischemic stroke	[20, 62, 167]
Givinostat (ITF2357)	Pan-HDACi [approved]	• Diminished astrocyte activation and microgliosis in the hippocampus of an animal model of traumatic brain injury. Also alleviated the loss of HSP70 involved in neuroprotection, countered neurodegeneration and improved neurobehavioral recovery	[133]
HDI-1	Class II HDAC inhibitor [investigational]	• Prevented the loss of tight junction proteins (occludin, claudin-5, ZO-1), decreased phosphorylated p65 unit of NF-κB and pro-inflammatory cytokines (IL-1β, TNF-α, IL-6), upregulated Keap1/Nrf2/ARE/HO1 and decreased ROS, in hCMEC/d3 submitted to ischemic stroke conditions	[168]
Panobinostat	Pan-HDACi [approved]	• Reduced inflammation and astroglial activation by downregulating the TLR2-MyD88-IRF5 signaling in EAE mice. Improved mitochondrial function and ameliorated oxidative stress by decreasing NOX2 and increasing Nrf2	[169]
RGFP966	HDAC3 inhibitor [investigational]	• Attenuated the loss of claudin-5 by increasing the acetylation of PPARγ in human BECs • A BBB repairing effect was observed through the preservation of junction proteins (claudin-5 and VE-cadherin) and activation of the miR-200a/Keap1/Nrf2 signaling pathway in an animal model of type 2 diabetes • Enhanced Nrf2 nuclear translocation and antioxidant effects were observed in an animal model of traumatic brain injury. Lower inflammation was obtained by inhibiting the HMGB1-TLR4-mediated activation of NLRP3 inflammasome. Also suppressed pro-inflammatory cytokines, and promoted higher acetylation of the p65 subunit of NF-κB in reactive astrocytes, decreasing its transcriptional activity • Inhibited the AIM2 inflammasome through higher STAT1 acetylation, in primary cultured microglia and in an animal model of stroke • Increased BDNF expression by preventing the deacetylation of H4K5ac, a binding site of BRD4. This enabled the binding of BRD4 to its gene promoter, improving learning and memory • Identical anti-inflammatory, antioxidant, and neuroprotective effects were observed in animal models of LPS-induced depressive-like behavior, neurodegenerative diseases, multiple sclerosis, surgical brain injury, and spinal cord injury	[59, 72, 81, 82, 95, 98– 101, 124,170– 173]
Romidepsin (FK228)	Class I HDAC inhibitor [approved]	• Decreased neuronal ferroptosis by activating the Keap1/Nrf2/HO1 antioxidant signaling pathway in microglia, in an animal model of intracerebral hemorrhage	[174]
Sodium butyrate	Pan-HDACi [investigational]	• Restored BBB integrity by increasing tight junction proteins (occludin, ZO-1) in animal models of depression and Parkinson’s disease. Also displayed antiapoptotic effects by increasing the Bcl-2/Bax ratio • Decreased the activation and number of microglia, and suppressed neuroinflammation through GPR109A, PPAR-γ, TLR4/MyD88/NF-κB signaling pathways, in animal models of ischemic stroke and alcohol-induced injury • Upregulated HSP70, while blocking p53, iNOS and COX-2, identically to trichostatin A and valproic acid • Enhanced BDNF in the hippocampus through the ACSS2/H3K9ac/BDNF pathway	[132, 175–180]
TMP269	Class II HDAC inhibitor [investigational]	• Counteracted BBB impairment by increasing the levels of tight junction proteins (occludin, claudin-5, ZO-1) in an animal model of ischemic stroke • Decreased microglia reactivity and upregulated neurotrophic factors, improving motor symptoms, in an animal model of Parkinson’s disease. Also enhanced synaptic plasticity and improved depressive symptoms by increasing the levels of BDNF, through HDAC5 inhibition, in a mouse model of depression	[73, 181, 182]
Trichostatin A	Class I and Class II HDAC inhibitor [investigational]	• Increased protection against oxidative stress in astrocytes through Nrf2 activation. Promoted the dissociation of Keap1:Nrf2 and Nrf2 acetylation, nuclear translocation, binding to antioxidant response element, and HO1 transcription	[132, 183, 184]
Tubastatin A	HDAC6 inhibitor [investigational]	• Ameliorated BBB dysfunction, decreasing the loss of tight junction proteins (occludin, ZO-1) • Exhibited neuroprotective effects by upregulating the expression of acetylated α-tubulin and inhibiting actin stress fiber formation, in an animal model of intracerebral hemorrhage	[185]
Valproic acid	Pan-HDACi [approved]	• Counteracted BBB dysfunction by decreasing MMP-9, tight junction degradation (claudin-5 and ZO-1), excessive NO production, and suppressing the nuclear translocation of NF-κB. Also demonstrated antiapoptotic effects, identically to sodium butyrate • Reduced microglial reactivity and enhanced the acetylation of the STAT1/NF-κB pathway through HDAC3 inhibition, in an animal model of spinal cord injury	[128, 132, 186–188]
Vorinostat (SAHA)	Pan-HDACi [approved]	• Ameliorated BBB leakage (lower IgG extravasation), neuroinflammation and oxidative stress, reducing the activation of cortical microglia, in an animal model of ischemic stroke • Induced the expression of P-glycoprotein in human BECs through the aryl hydrocarbon receptor • Protected astrocytes from INF-γ toxicity by decreasing STAT3 phosphorylation • Attenuated microglia reactivity by inhibiting the TLR4/MyD88/NF-κB pathway, in an animal model of epilepsy. Also elicited identical effects to those of entinostat, regarding the microglia regulator MITF • Demonstrated neuroprotection by promoting the release of neurotrophic factors (GDNF and BDNF) from astroglia through histone hyperacetylation	[70, 85, 167, 189–191]
W2A-16	Class I HDAC inhibitor [investigational]	• Enhanced BBB integrity by inhibiting class I HDACs in hCMEC/D3 cells and human induced pluripotent stem cell (iPSC)-derived BECs. Increased the expression of tight junction proteins (ZO-1 and occludin) and adherens junction proteins (VE-cadherin) • Activated Wnt/β-catenin signaling, decreased neuroinflammation, and attenuated amyloid and tau phosphorylation in 5xFAD mice, PS19 mice and iPSC-derived cerebral organoids from Alzheimer’s disease patients	[71, 192]
Other modulators			
APC	CBP inhibitor [approved]	• Through EPCR-dependent and PAR1‐dependent signaling, APC counteracted LPS-mediated HMGB1 secretion by downregulating the interaction of CBP with HMGB1, and the translocation of HDAC2 and HDAC4 out of the nucleus	[193]
Dimethyl fumarate	Class I and II HDACs inhibitor [approved]	• Reduced class I and II HDAC mRNA levels, HDAC protein levels, and HDAC activity, induced by inflammatory cytokines (IL-1β, TNF-α, IFNγ) in primary rat astrocytes. Also increased the acetylation levels of non-histone proteins (i.e., ADP/ATP translocase 2, core histone macro-H2A.1, formin-binding protein 1, phosphatidylinositol-3,4,5-trisphosphate 5-phosphatase 1, phosphatidylinositol-binding clathrin assembly protein, thymosin beta-4) • Increased Nrf2 and induced HO1, identically to vorinostat	[134]
Fenofibrate	SIRT1 activator [approved]	• Exhibited an anti-inflammatory effect on microglia by suppressing NF-κB, likely through SIRT1-mediated deacetylation	[130]
Fluoxetine	HDAC4 inhibitor [approved]	• Attenuated BBB dysfunction by mitigating MMP-9 activity, the degradation of tight junction proteins and expression of pro-inflammatory cytokines. This was mediated by a decrease of TLR4/MyD88/NF-κB signaling pathway • In adult rats, fluoxetine decreased HDAC4 hippocampal overexpression, associated with depression-like behavior. It prevented HDAC4 recruitment to *mTOR* and *Gnai1* promoters, reducing the decline in their expression. Depression-like and anxiety-like behaviors were normalized • Post-natal fluoxetine-induced HDAC4 increase and mTOR decline were countered by sodium butyrate, which prevented the emergence of depression- and anxiety-like behaviors	[194, 195]
Leonurine (SCM-198)	HDAC4 activator [investigational]	• Protected BBB integrity by increasing tight junction proteins (occludin, claudin-5, ZO-1) in an animal model of ischemic stroke. This was caused by higher HDAC4 expression, which activated the HDAC4/NOX4/MMP-9 pathway, inhibiting MMP-9	[61]
Lithium	HDAC1 inhibitor [approved]	• Alleviated BBB dysfunction due to the activation of endothelial Wnt/β-catenin signaling, in animal models of depressive behavior. Besides acting as a GSK3-β inhibitor, lithium also inhibited HDAC1, a repressor of β-catenin, leading to the upregulation of the Wnt/β-catenin pathway • Restored depressed histone acetylation caused by reactive microglia, and reinstated Nrf2-mediated transcription and antioxidant defense in astrocytes	[184, 196, 197]
Melatonin	SIRT1 activator [approved]	• Preserved BBB integrity by reducing MMP-9 and increasing tight junction expression (claudin-5, occludin, and ZO-1), which resulted in improved cognitive function, in an animal model of chronic cerebral hypoxia • Activated the SIRT1/PGC-1α/PPARγ signaling pathway, leading to lower NF-κB and NLRP3 inflammasome	[131]
Memantine	HDAC inhibitor [approved]	• Exhibited neuroprotective effects due to inhibited HDAC activity, which triggered the release of GDNF from astrocytes through histone hyperacetylation on *gdnf* promoter • Also lowered LPS-induced microglia reactivity and displayed anti-inflammatory and antioxidant effects, decreasing ROS, TNF-α, prostaglandin E_2_ and NOS	[198]
Perampanel	SIRT3 activator [approved]	• Preserved BBB function through SIRT3 upregulation after traumatic neuronal injury or excitotoxicity, decreasing oxidative stress and neuroinflammation (IL-1β and IL-6)	[199]
Rosiglitazone	SIRT1, SIRT6 activator [approved]	• Ameliorated the loss of BBB integrity induced by tobacco smoke, by increasing tight junction proteins (occludin, claudin-5, ZO-1). Also exhibited anti-inflammatory and antioxidant effects by upregulating PPARγ and Nrf2/ARE/HO1, and reducing NF-κB and pro-inflammatory cytokines (TNF-α, IL-6) • Increased SIRT1/PGC-1α/PPARγ in N2A cells, enhancing mitochondrial function. Also augmented SIRT6 levels and rescued BNDF loss in a mouse model of Huntington’s disease	[129, 200, 201]
Resveratrol	SIRT1 activator [investigational]	• As a SIRT1 activator, attenuated neuronal damage and neurological dysfunction, in an animal model of EAE. Also displayed anti-inflammatory, antioxidant and BBB protecting properties, preventing the loss of tight junction proteins (occludin, claudin-5, ZO-1) and suppressing pro-inflammatory cytokines (iNOS, IL-1β) • In combination with entinostat, revealed neuroprotection in a mouse model of cerebral ischemia, and primary cortical neurons exposed to oxygen-glucose-deprivation. Neurological deficits and microglia inflammatory response were reduced	[202–204]
Rutin	HDAC1 inhibitor [investigational]	• Alleviated BBB dysfunction by preventing HDAC1 from restricting the access to the *claudin-5* promoter and stabilizing H3K27ac, which rescued the expression of claudin-5 in the hippocampus. This decreased the passage of TNF-α into the hippocampus and ameliorated cognitive impairment, in a mouse model of depression	[69]

ACSS2, acetyl-CoA synthetase 2; APC, activated protein C; ARE, antioxidant responsive elements; BDNF, brain-derived neurotrophic factor; BECs, brain endothelial cells; BRD4, bromodomain-containing protein 4; CBP, CREB-binding protein; CREB, cAMP-response element binding protein; EAE, Experimental autoimmune encephalomyelitis; EPCR, endothelial protein C receptor; GDNF, glial derived neurotrophic factor; GPR109A, hydroxycarboxylic acid receptor 2, IL, interleukin; iNOS, inducible nitric oxide synthase; LPS, lipopolysaccharide; GNAI1, guanine nucleotide-binding protein alpha inhibiting 1; GSK3-β, glycogen synthase kinase 3 beta; HDAC, histone deacetylase; HMGB1, high mobility group box 1 protein; HO1, heme oxygenase 1; IFN-γ, interferon gamma; IL, interleukin; IRF5, interferon regulatory factor 5; Keap1, Kelch-like ECH-associated protein 1; MCP-1, monocyte chemoattractant protein-1; MITF, microphthalmia-associated transcription factor; mTOR, mammalian target of rapamycin; MyD88, myeloid differentiation primary response 88; NF-κB, nuclear factor-kappa B; NOX, NADPH oxidase; Nrf2, nuclear factor erythroid-2 related factor 2; NT-3, neurotrophin-3; PAR1, protease-activated receptor 1; PGC-1α, peroxisome proliferator-activated receptor-gamma coactivator 1-alpha; PPARγ, peroxisome proliferator-activated receptor-gamma; ROS, reactive oxygen species; SIRT, sirtuin; TNF-α, tumor necrosis factor-α; TLR, toll-like receptor

HDAC inhibitors are epigenetic-based therapies, mostly applied for the treatment of cancer malignancies. The Food and Drug Administration (FDA) approved romidepsin and vorinostat (cutaneous T-cell lymphoma) and belinostat (peripheral T cell lymphoma), while the European Medicines Agency (EMA) approved panobinostat (multiple myeloma). In parallel, givinostat was approved by both agencies for Duchenne muscular dystrophy [47, 48]. Several HDAC inhibitors are under different stages of clinical trials, including for CNS therapeutic indications, such as neurodegenerative diseases. Others are currently used only for research purposes [41, 49]. Based on their chemical structure, HDAC inhibitors can be divided into hydroxamic acids (e.g., belinostat, panobinostat, and vorinostat); short-chain fatty acids (e.g., valproic acid and sodium butyrate); cyclic peptides (romidepsin) and benzamides [47, 50]. At the present moment, valproic acid is the only HDAC inhibitor approved for CNS diseases, namely epilepsy, bipolar disorder, and migraine prophylaxis. Most marketed HDAC inhibitors are non-selective (or pan) inhibitors that do not target specific HDAC isozymes, but rather HDACs from different classes. Moving toward isozyme-selective HDAC inhibitors was suggested to reduce adverse effects attributed to a lack of selectivity [41, 47, 51, 52].

Building upon the overview that neurobiological functions are affected by epigeneticregulation, including acetylation and deacetylation processes, it becomes relevant toinvestigate how HATs/HDACs modulate the function of distinct cellular components of the BBB. Since its function and integrity depend heavily on the coordinated behavior of BECs, astrocytes, pericytes, and microglia, understanding how epigenetic marks influence these cells can provide insight into both physiological regulation and pathological modifications of the BBB. Hence, in the following sections, key acetylation and deacetylation epigenetic mechanisms in the several cells responsible for the regulation of BBB function will be analyzed.

## HAT/HDAC modulation of brain endothelial cells

Lining cerebral blood vessels, BECs are one of the first points of contact with the BBB, essential for maintaining CNS homeostasis. BECs regulate both transcellular and paracellular routes of transport through the selective expression of tight junction proteins [e.g., claudin-5, occludin, zonula occludens-1 (ZO-1), and junctional adhesion molecules] and adherens junction proteins [e.g., vascular endothelial (VE-) cadherins], influx and efflux transporters (e.g., P-glycoprotein and Breast Cancer Resistance Protein), and metabolic cytochrome P450 (CYP) enzymes, reflecting the CNS requirements for a tightly regulated ionic and molecular environment [5, 10, 53].

The epigenetic modification of histones and chromatin-modifying enzymes through HDAC2 activity is critical for the transcriptional regulation of BBB genes, and BBB maturation. During development, active Wnt signaling enhances the expression of genes necessary for BBB formation. However, when Wnt signaling decreases, repressors HDAC2 and polycomb repressive complex 2 (PRC2) are recruited to BBB genes, enabling BBB stabilization and maintenance. Hence, in adult BECs, there is low Wnt signaling and Wnt target genes are transcriptionally repressed due to the presence of HDAC2 and PRC2. In agreement, HDAC2 inhibition with trichostatin A (pan-inhibitor) and MS-275 (class I inhibitor) promoted the re‑expression of genes that were originally active during developmental BBB formation, offering an opportunity for therapeutic intervention in case of BBB dysfunction [54].

Other findings highlight the importance of histone acetylation in maintaining endothelial stability and BBB integrity. For instance, the Nogo-B receptor (NgBR), expressed on the surface of BECs, modulates the expression of cerebral cavernous malformation (CCM) genes, *CCM1* and *CCM2*, through a HAT from the MYST family, known as histone acetyltransferase binding to ORC1 (HBO1; MYST2) [55]. Through NgBR activation, HBO1 mediates the acetylation of histone H4 at lysines 5 and 12 (H4K5 and H4K12), enhancing the transcription of CCM1 and CCM2. Then, the CCM1/2 complex stabilizes endothelial intercellular junctions by promoting VE-cadherin-mediated adherens junctions; reducing cytoskeletal tension; and preventing the aberrant formation of cell extracellular matrix adhesions (e.g., focal adhesions), which anchor BECs to the basement membrane [56, 57]. In NgBR-deficient human BECs, the impaired recruitment of HBO1 to CCM1/2 promoters resulted in reduced histone acetylation and gene expression. This epigenetic alteration led to increased Ras homolog gene family member A (RhoA) / Rho associated kinase (ROCK) signaling, a pathway responsible for the assembly and disassembly of tight junctions. RhoA/ROCK activation mediates the phosphorylation of myosin II regulatory light chain (MLC2), which destabilizes tight junction proteins through the contraction of the perijunctional actomyosin ring [58]. Hence, RhoA/ROCK/MLC2 increases endothelial contractility, impairs tight junction proteins, and triggers BBB hyperpermeability. In the study of Fang et al. [55], structural defects were observed in mice with endothelial-specific NgBR knockout. Restoring HBO1 expression rescued CCM1/2 transcription and BBB integrity, underscoring the role of HBO1-mediated histone acetylation in the epigenetic regulation of tight junctions and cerebrovascular homeostasis [55].

Additional examples highlight the broader role of distinct HDAC isozymes in BBB regulation through the modulation of endothelial function. However, it should be mentioned that the roles of individual HDAC isozymes are context-dependent. For instance, under oxygen-glucose deprivation and reoxygenation (OGD/R) conditions, the increased expression of HDAC3 and HDAC9 has been linked to enhanced endothelial permeability and disruption of tight junctions. These effects were reversed by isozyme-specific inhibition of HDAC3 by RGFP966 in primary human BECs [59, 60]. Conversely, HDAC1 and HDAC4 demonstrated protective effects in vitro (OGD/R) and also in in vivo models of ischemia/reperfusion (I/R) [61, 62].

In alignment with the beneficial effects described for HDAC1, another study suggested that, at a basal level of activity, HDAC1 may prevent BECs from entering senescence. In this study, ionized radiation-treated human and mouse BECs exhibited reduced HDAC1 expression [63]. This reduction contributed to cellular senescence through mitochondrial dysfunction, both of which are proposed as hallmarks of aging and closely related to neurodegenerative diseases such as Alzheimer’s disease [64, 65]. Specifically, HDAC1 knockdown resulted in a decreased expression of SIRT1, a class III HDAC, increasing the acetylation (and inactivation) of the mitochondrial regulator PPAR coactivator (PGC)-1α. Given that PGC-1α is a central activator of mitochondrial function and biogenesis genes, and that its acetylation promotes a dissociation from target gene promoters, these modifications likely contributed to the observed mitochondrial impairment [66–68]. In agreement, the downregulation of PGC-1α was shown to reduce endothelial barrier integrity in BECs, suggesting that impaired mitochondrial function contributes to endothelial cell senescence. While SIRT1 is known to deacetylate PGC-1α directly, the extent to which HDAC1 influences mitochondrial function through SIRT1 regulation versus direct interaction with PGC-1α, remains unclear [63].

Nevertheless, the role of HDAC1 is different in depression, accentuating its context-dependent effect, according to cellular localization, non-histone deacetylation and interacting partners [42, 46]. Dudek et al. [20] identified an upregulation of HDAC1 in mice susceptible to chronic social defeat stress, as well as in the nucleus accumbens of depressed patients. It was demonstrated that endothelial inflammation mediated by tumor necrosis factor-α (TNF-α)/NF-κB led to HDAC1 recruitment, which prevented access to the promoter gene of *claudin-5*, thereby causing transcriptional repression, claudin-5 loss and BBB hyperpermeability. HDAC1 inhibition by MS-275 enhanced social interactions and rescued claudin-5 expression [20]. Identically, Sun et al. [69] evidenced that HDAC1 inhibition by the flavonoid rutin has a protective effect on the BBB integrity of mice exposed to unpredictable chronic mild stress (Table 2). Reinstating claudin-5 decreased the access of TNF-α into the hippocampus and ameliorated cognitive impairment [69].

In spite of the advances in the area, several notable limitations should be acknowledged. Many experiments have relied solely on BEC lines to model the BBB, which do not fully replicate the in vivo environment where mural cells and glial cells have key functions [63, 70–72]. Additionally, the lack of in vivo studies limits the understanding of the safety and effectiveness of HDAC inhibitors in complex physiological settings. While HDAC inhibitors are known to induce cell cycle arrest and apoptosis in cancer cells, this effect raises concerns about their use in non-cancer therapeutic contexts [59, 71, 73]. Furthermore, the non-selective nature of several HDAC inhibitors complicates the interpretation of experimental findings and their application in therapeutic practice, since different HDAC isozymes have distinct and sometimes opposing biological activities. These limitations highlight the need for comprehensive and physiologically relevant experimental approaches in future research, including further in vivo studies, in vitro co-cultures of BECs and other BBB elements, as well as 3D models, in a dynamic configuration.

## HAT/HDAC regulation of astrocytes and microglia

Glial cells, which make up most of the cells in the CNS, are broadly divided into two main groups: macroglia, including astrocytes and oligodendrocytes, and microglia [74]. The following section explores how acetylation and deacetylation mediate the functions of astrocytes and microglia at the BBB.

As previously mentioned, microglia are the resident macrophages of the CNS and participate in synaptic remodeling and the clearance of apoptotic cells, cellular debris (e.g., myelin debris), and toxic protein aggregates (e.g., α-synuclein). They also promote myelination by supporting the development and differentiation of oligodendrocytes [16, 74–76]. In response to injury or infection, microglia rapidly release a variety of immune mediators, including pro- and anti-inflammatory cytokines, chemokines, and complement proteins [77].

Microglial reactivity is also affected by epigenetic modifications (Table 2). In the context of the intervention of epigenetics in glial inflammation, it has been demonstrated that an epigenetic axis exists in a mouse model of multiple sclerosis [experimental autoimmune encephalomyelitis (EAE)]. This axis is composed by a lysine specific demethylase 1 (LSD1) that interacts with a repressive transcription complex, formed by REST corepressor 1 (RCOR1) and HDAC1/2. The inhibition of the LSD1/RCOR1/HDAC axis with vafidemstat resulted in the induction of gene expression with neuroprotective effects, namely the inhibition of demyelination and a reduction of microglial activation [78]. Dey et al. [79] showed that microglia contribute to the pathogenesis of EAE through a chromatin reader that binds to acetylated histones, known as bromodomain-containing protein 4 (BRD4), needed for microglia function and transcriptome. BRD4 deletion in microglia decreased EAE severity through lower demyelination and neuroinflammation [79].

In microglia exposed to LPS, activation of the toll-like receptor 4 (TLR4) signaling pathway has been found to upregulate the expression of the lysine acetyltransferase general control of amino acid synthesis 5 (GCN5), also known as KAT2A. GCN5 modulates the acetylation of p65 and allows the nuclear translocation and transcriptional activity of NF-κB, triggering a pro-inflammatory response. Hence, it was suggested that GCN5 inhibition may be explored as a strategy to mitigate neuroinflammation [80].

HDAC3 expression is also upregulated in microglia, in response to cellular damage [81, 82]. HDAC3 appears to promote a pro-inflammatory response, as its knockout in microglia of mouse models of brain injury [83] and stroke [84], as well as its pharmacological inhibition in animal models of depression, stroke, and spinal cord injury (Table 2) attenuated inflammation, conferred neuroprotection and stimulated remyelination. Meleady et al. [85] and Huang et al. [86] investigated how HDAC3 affects microglial cell immune responses and immune memory, following acute exposure to LPS and exposure to LPS followed by a second insult (priming), respectively. In the first case, HDAC3 inhibition with RGFP966, vorinostat or romidepsin, improved microglial phagocytic capacity and reduced the expression of arginase 1 (Arg1). Since Arg1 competes with inducible nitric oxide synthase (iNOS) for L-arginine, its sustained expression may shift microglial activity away from excessive nitric oxide production, known to be cytotoxic [85, 87, 88]. Furthermore, HDAC3 inhibition increased both global and promoter-specific histone acetylation marks, H3K27ac and H3K9ac. It was evidenced that HDAC3 inhibition enhances the neuroprotective and immunomodulatory functions of microglia under inflammatory conditions [85]. In the second case, it was demonstrated that priming microglia with LPS exacerbated the inflammatory response to a secondary stressor, manganese, through increased H3K27ac deposition. Indeed, accumulating evidence suggests that immune memory shaped by environmental stimuli is epigenetically stored through specific enrichment of H3K27ac, particularly in microglia [86, 89, 90]. H3K27ac was identified as a key epigenetic mark associated with the formation of this innate immune memory. Reducing H3K27ac levels by targeting the histone acetyltransferases p300/CBP, responsible for catalyzing H3K27 acetylation at enhancer regions, with a selective inhibitor (GNE-049), led to the suppression of iNOS expression and downstream inflammation, implicating H3K27ac as a molecular driver of the pro-inflammatory response. Overall, these findings highlight the role of H3K27ac and p300/CBP in modulating microglial immune memory [86, 91]. Although the previous studies may initially appear conflicting, it should be noted that distinct contexts of microglial activation and epigenetic modulation are described. In the absence of priming, HDAC3 inhibition reduces microglial reactivity; however, following a previous exposure to an inflammatory stimulus, lasting acetylation marks can predispose microglia to trigger a stronger pro-inflammatory response, upon a subsequent insult.

On the other hand, astrocytes are star-shaped, highly branched cells, strategically located between neurons and BECs [53, 92]. These cells mediate signals between neurons and the vasculature through their end-feet projections, dynamically modulating the levels of water, ions, neurotransmitters, and CBF. These glial cells also modulate BBB properties through the secretion of paracrine factors, which can either increase [e.g., vascular endothelial growth factor (VEGF), nitric oxide and MMPs] or decrease (e.g., sonic hedgehog, retinoic acid and angiopoietin-1) its permeability. For instance, the secretion of the ligand sonic hedgehog enhances BBB integrity by binding to the transmembrane receptor Patched-1 on the surface of BECs, leading to the induction of tight junction proteins, such as ZO-1 and occludin; the downregulation of intercellular adhesion molecule, thereby limiting unnecessary immune cell adhesion and transendothelial migration; and the reduction of pro-inflammatory cytokine release [e.g., interleukin (IL)-6, IL-1β, and TNF-α] by microglia. Conversely, reactive astrocytes can impair BBB integrity by secreting MMPs, such as MMP-2 and MMP-9, that degrade extracellular matrix components and tight junction proteins, as well as VEGF, which promotes the downregulation and disruption of intercellular junctions [1, 10, 93, 94].

Aquaporin-4 (AQP4) is a water channel protein predominantly located in the end-feet of astrocytes, where it surrounds blood vessels and regulates brain water homeostasis [1, 10]. Under ischemic conditions, AQP4 has been proposed to be upregulated, contributing to astrocyte swelling, cerebral edema, and BBB leakage [95, 96]. It has been observed that epigenetic mechanisms influence this dysregulation process. In a rat model of I/R injury, it was demonstrated that selective inhibition of HDAC3 significantly reduced AQP4 expression in glial fibrillary acidic protein (GFAP)-positive astrocytes [95]. Notably, HDAC3 inhibition also reduced the immunoreactivity of the ionized calcium-binding adapter molecule 1, a protein expressed in microglia, suggesting a decrease in microglial responsiveness to I/R injury. These cellular changes were associated with improved cerebral edema and lower BBB leakage [95, 97]. Beneficial effects of RGFP966-mediated HDAC3 inhibition on astrocyte immunoreactivity were also observed in the context of neurodegenerative diseases, promoting neuroprotection, learning and memory [98–101].

These findings support the overall deleterious role of HDAC3 in modulating astrocyte reactivity and cognitive dysfunction.

The activation of a pro-inflammatory response in astrocytes through NF-κB is also mediated by epigenetic mechanisms [102]. NF-κB is thought to mediate epigenetic changes through its ability to recruit corepressors (e.g., HDAC1) or coactivators with HAT activity (e.g., CBP and p300) depending on its phosphorylation state [103, 104]. The p50-HDAC-1 complexes decrease NF-κB-dependent gene expression, whereas NF-κB complexes containing phosphorylated p65, associate with CBP, displace the p50-HDAC-1 complexes and activate transcription [103]. Consistent with previous studies, an interaction with coactivators CBP/p300 enhances histone acetylation at target gene promoters, facilitating transcriptional activation and sustaining pro-inflammatory gene expression [102, 105, 106]. This interaction was associated with increased acetylation of histone residues H3K9, H3K14, and H3K27 in astrocytes. Importantly, H3K27 acetylation was also observed in vivo in GFAP-positive astrocytes from the ischemic penumbra of rat brains [102]. Although this study does not directly examine BBB integrity, astrocytes exhibiting a reactive, proinflammatory phenotype, characterized by NF-κB activation, were shown to drive brain inflammation and contribute to BBB dysfunction [38].

Lastly, as previously noted in the context of BECs, HDAC1 plays a protective role regarding astrocyte and microglia reactivity in ischemic conditions. In this case, HDAC1 inhibition by MS-275 was detrimental and exacerbated gliosis in both astrocytes and microglia, further supporting HDAC1-mediated neuroprotection in stroke [62]. In agreement, Pao et al. [107] revealed that HDAC1 is beneficial against brain aging and neurodegeneration. HDAC1 deletion in mouse astrocytes and neurons resulted in astrogliosis, higher GFAP immunoreactivity, and astrocytic hypertrophy. Furthermore, *Hdac1* cKO mice demonstrated age-dependent cognitive decline. The pharmacological activation of HDAC1 with exifone improved the memory and cognition of mice [107].

Notwithstanding, several limitations emerge from the previous studies. Many experiments have relied on microglial cell lines, which, while useful for controlled in vitro analyses, do not fully capture the regulatory complexity of in vivo microglia [85]. Additionally, the field currently lacks epigenomic tools capable of precisely targeting specific combinations of epigenetic marks, and there is still a limited understanding of how these marks interact to regulate gene expression [86]. Thirdly, molecular targets of HDAC inhibitors remain incompletely defined, as these molecules modulate not only histone proteins but also non-histone substrates [102]. Their selectivity towards specific classes of histones is sometimes less stringent than expected, hindering the interpretation of their function in brain tissue [62, 102].

## HAT/HDAC impact on pericytes

As aforementioned, pericytes share a basement membrane with BECs to which they are connected via peg-and-socket junctions, ensuring the maintenance of BBB integrity. Beyond structural support, pericytes promote angiogenesis and BEC proliferation, secrete extracellular matrix, and clear debris. Pericytes are also involved in neurovascular coupling, integrating neurovascular signals and controlling CBF through vessel contractility. Additionally, growing evidence suggests that pericytes can transform into multipotent stem cells capable of differentiating into a variety of cell types, including those of neural and vascular lineages [108]. Among the BBB elements herein discussed, pericytes have the least amount of data on acetylation- and deacetylation-based epigenetic regulatory mechanisms, indicating that additional research is needed.

In the study of Loan et al. [109], acetylation and deacetylation mechanisms were identified as key regulators of the neurogenic potential of nerve/glial antigen 2 (NG2)-expressing pericytes in response to ischemic stroke. NG2, also known as chondroitin sulfate proteoglycan-4, serves as a transmembrane proteoglycan that interacts with both extracellular ligands and the cytoskeleton. It was demonstrated that NG2 + pericytes generate radial glial precursors at the lesion site, which contribute to neural regeneration. Central to the regulation of this pericyte-to-neuron reprogramming process is the activity of the histone acetyltransferase CBP, whose phosphorylation at serine 436 directs an acetylation shift between the transcription factor Sex-determining Region Y-Box 2 (Sox2) and histone H2B. When phosphorylated, CBP preferentially acetylates Sox2, promoting its nuclear localization and transcriptional activity, while reducing H2B acetylation, in a competitive process [109]. These findings underscore the importance of dynamic acetylation regulation in modulating NG2 + pericyte plasticity and guiding their differentiation into neural lineages following injury, a process that may be tightly linked to the preservation or restoration of BBB integrity in the post-ischemic environment [109]. One limitation of the study is that it does not show whether the newly generated neurons from pericytes functionally integrate into brain circuits or contribute to recovery after stroke. Additionally, while human pericytes were successfully reprogrammed in vitro, it remains uncertain whether this process can occur naturally or be safely induced in the human brain after injury.

In another study, Dave et al. [110] investigated the participation of pericytes in the regulation of BEC behavior in germinal matrix hemorrhage, a condition that may affect neonates. The authors evidenced that transforming growth factor-β (TGF-β), which mediates BEC-pericyte crosstalk, induces the repression of angiopoietin-2 (Angpt2) through HDAC5 [110]. Angpt2 is an endothelial growth factor known to promote the loss of microvascular integrity, leading to vascular leakage under pathological conditions of inflammation, ischemia, and tumor angiogenesis by synergy with VEGF [111, 112]. When HDAC5 is recruited to the *Angpt2* promoter, it causes its deacetylation and transcriptional epigenetic silencing. However, in cases of germinal matrix hemorrhage, there is an upregulation of Angpt2 due to the loss of repression. The pharmacological or genetic inhibition of pericyte Angpt2, with resort to an anti-Angpt2 antibody or through *Angpt2* gene deletion, resulted in the reduction of germinal matrix hemorrhage, normalized vessel morphology, and decreased BEC hyperproliferation. Thus, TGF-β-mediated repression of pericyte-derived Angpt2, via HDAC5, is important for BBB integrity and may be a target against germinal matrix hemorrhage [110].

Lastly, Sheikh et al. [113] demonstrated that the depletion of males absent on the first (MOF), also known as MYST1 or KAT8, in neural cells, led to a loss of H4K16 acetylation, which alters neural metabolic environment, and prompts the accumulation of long-chain fatty acids. Pericytes respond to this by activating the TLR4/myeloid differentiation primary response 88 (MYD88)/NF-κB pathway, resulting in a pro-inflammatory response, dysfunction of brain microvasculature and hemorrhage. In opposition, although pericytes responded to this trigger, BECs remained unaffected [113]. In sum, it can be observed that histone acetylation (CBP, MOF) or deacetylation (HDAC5) affect the participation of brain pericytes in processes of neural regeneration and metabolism, influencing neurovascular integrity.

**Fig. 2:**
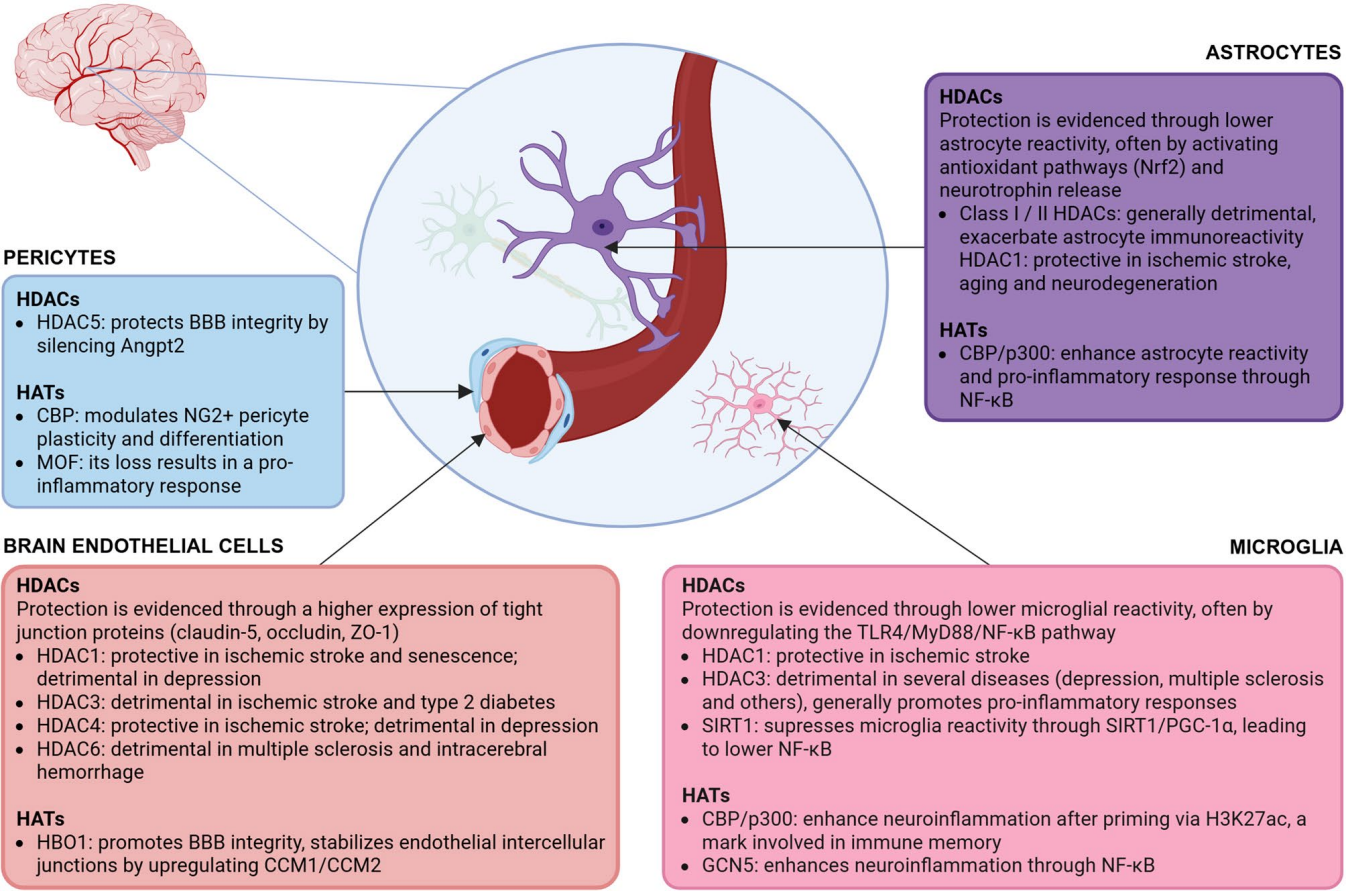
Influence of epigenetic regulation on blood-brain barrier (BBB) integrity and function mediated by histone acetyltransferases (HATs) and histone deacetylases (HDACs). Angpt2, angiopoietin-2; CBP/p300, CREB binding protein/E1A-associated protein p300; GCN5, general control of amino acid synthesis 5; HBO1, histone acetyltransferase binding to ORC1; MOF, males absent on the first; MyD88, myeloid differentiation primary response 88; NF-κB, nuclear factor kappa B; Nrf2, nuclear factor erythroid-2 related factor 2; PGC-1α, peroxisome proliferator-activated receptor-gamma coactivator 1-alpha; PPARγ, peroxisome proliferator-activated receptor-gamma; SIRT1, sirtuin 1; TLR4, toll-like receptor 4; ZO-1, zonula occludens-1. Elaborated in BioRender^®^

Figure 2 summarizes the overall impact of acetylation and deacetylation on BBB integrity through the activity of HATs and HDACs isozymes in BECs, pericytes, astrocytes and microglia.

## Influence of epigenetic modulators on blood-brain barrier impairment

In recent years, several studies emerged describing the BBB repairing capacity of a diverse range of molecules with consequent amelioration of disease symptoms and neuroprotection. This has been demonstrated through the increase of tight junction protein expression between BECs, and through antioxidant and anti-inflammatory effects in astrocytes and microglia (Fig. 2; Table 2). While BBB repair has been directly related with non-selective (pan) and selective HDAC inhibitors, this connection remains less clear in the case of HAT activators, requiring further investigation. For instance, CTPB and TTK21 are p300/CBP activators that enhance histone acetylation and promote synaptic plasticity in models of spinal cord injury [114–116], Alzheimer’s disease [117–120], Parkinson’s disease [121], and neurodevelopmental disorders [122, 123]. Nevertheless, their impact on BBB impairment remains unexplored.

Common pathways responsible for the anti-inflammatory and antioxidant effects of HDAC inhibition are the TLR2(4)/MyD88/NF-κB pathway and the Kelch-like ECH-associated protein 1 (Keap1)/ nuclear factor erythroid-2 related factor 2 (Nrf2)/ antioxidant responsive elements (ARE)/ heme oxygenase 1 (HO1) pathway, respectively. These effects are not only observed with investigational and clinically approved HDAC inhibitors, but also with other molecules through HDAC modulation (Table 2). Although the use of pan-HDAC inhibitors complicates the understanding of the individual contribution of each isozyme, the selective inhibition of HDAC3 with RGFP966 revealed that this isozyme is, at least in part, responsible for pathological astrocyte reactivity [100], and microglial reactivity through the HDAC3/TLR4/ NOD-, LRR- and pyrin domain-containing protein 3 (NLRP3) signaling pathway [124]. Class I and class II HDAC inhibition with TSA and dimethyl fumarate also enhanced the release of Nrf2 by astrocytes, and decreased the release of pro-inflammatory mediators. Interestingly, sodium phenylbutyrate, vorinostat and memantine promoted the release of neurotrophins [brain-derived neurotrophic factor (BDNF), neurotrophin-3 (NT-3), glial derived neurotrophic factor GDNF] from astrocytes (Table 2).

As aforementioned, in addition to histones, HATs/HDACs (de)acetylase non-histone substrates, encompassing transcription factors, cytoskeletal proteins, and molecular chaperones, elegantly summarized by Kabir et al. [40] and Wang et al. [44]. These include, but are not limited to HDAC1/2/4-mediated deacetylation of p53 [49, 125, 126] and HDAC3 inhibition/SIRT1 activation-mediated effects on NF-κB [100, 127–131]. Sodium butyrate, trichostatin A and valproic acid are known to repress the transcription factor and proapoptotic protein p53, in cerebral ischemia, eliciting neuroprotective effects [132]. On the other hand, NF-κB regulation by acetylation is highly complex, depending on cell type and on which lysine residues are modified [104]. For instance, belinostat, RGFP966 and valproic acid inhibited HDAC3, increased the acetylation of p65, and suppressed NF-κB activation in astrocytes and microglia. In contrast, fenofibrate, melatonin and rosiglitazone inhibited NF-κB through SIRT1 activation, and deacetylation (Table 2). Among chaperones, givinostat, sodium butyrate, valproic acid and trichostatin A, upregulated Hsp70, resulting in neuroprotection [132, 133]. Dimethyl fumarate, a class I and II HDACs inhibitor, also promoted the deacetylation of non-histone proteins, influencing their neuroprotective or neurotoxic functions (Table 2). These include ADP/ATP translocase-2, a mitochondrial membrane protein increased in reactive astrocytes; formin binding protein 1, implicated in glial cell activation; and thymosin beta-4, shown to inhibit astrocyte apoptosis [134].

Lastly, challenges remain regarding the delivery of HDAC inhibitors to the CNS, and their neuropharmacokinetics. Reported issues include short half-life times in humans [e.g., belinostat (1.1–2.9 h), vorinostat (2 h), romidepsin (< 3 h), givinostat (6–6.9 h)] with exceptions, such as panobinostat (14.6–17.1 h) and entinostat (39–80 h) [135–140]; limited access to the CNS in non-human primates (e.g., belinostat, entinostat, panobinostat) [141–143]; inter-patient variability [139, 144]; and drug-drug interactions mediated by CYP enzymes and efflux transporters, identified for vorinostat [70, 145], romidepsin [137], panobinostat [139] and valproic acid [70]. For instance, vorinostat upregulated the expression of P-glycoprotein and CYP1A1 in human BECs by increasing the binding of acetylated histone H3K9/K14 and aryl hydrocarbon receptor (AHR) proteins to regions of the *ABCB1* and *CYP1A1* promoters, where AHR response elements are located [70]. It has also been evidenced that HDAC inhibitors modulate the expression P-glycoprotein and Breast Cancer Resistance Protein, which can have repercussions on CNS delivery, since both constitute a highly effective and cooperative efflux system at the BBB [146, 147]. HDAC inhibitors can either upregulate or repress these transporters, depending on cell and tissue type, molecular environment, disease conditions and duration of treatment, as well potency (e.g., more potent hydroxamic acids *versus* less potent short-chain fatty acids), and toxicity of the compounds. Since efflux transporters influence pharmacokinetics and drug-drug interactions, it is recommended to evaluate the effect of HDAC inhibitors in organs involved in drug disposition, and potentially relevant interactions with co-administered drugs across different genetic backgrounds, ages and comorbidities [146]. Although efflux can compromise CNS exposure, it may also be an opportunity in neurological conditions involving a compromised expression of efflux transporters and the accumulation of neurotoxicants (e.g., Alzheimer’s disease), in which case, the clinical use of HDAC inhibitors may be a relevant therapeutic approach to promote their clearance from the CNS [70, 148, 149].

An in-depth investigation of the neuropharmacokinetics of HDAC inhibitors is needed, encompassing rate, extent and intra-brain distribution. In mice, unbound brain-to-plasma ratios (K_p, uu_) of 0.06 ± 0.02 and 0.02 ± 0.001 were reported for vorinostat and quisinostat, respectively [150]. Data in non-human primates has been estimated resorting to CSF as a surrogate of brain exposure [141, 142], which requires caution, since unbound CSF concentrations may not necessarily reflect unbound brain concentrations [151]. Other authors have used positron emission tomography (PET), a powerful technique with increased translational potential [143]. Observed interspecies differences in brain exposure (rodents *versus* non-human primates and humans), possibly justified by differences in transporter expression at the BBB, call for the determination of relevant parameters, namely K_p, uu_, in higher species, and their careful interpretation [152]. These data, conjugated with potency, should be used to overcome translational difficulties and assist drug discovery.

## Conclusions

Epigenetic modifications unveil the dynamic nature of DNA to achieve a precise regulation of gene expression in response to various stimuli. In the CNS, these mechanisms take part in modulating the structural integrity and function of the BBB.

The purpose of this review was to examine how acetylation and deacetylation, two well-known epigenetic mechanisms governed by HATs/KATs and HDACs/KDACs, regulate gene expression in BBB-associated cells, including BECs, astrocytes, microglia, and pericytes. By exploring how these enzymes influence cell-specific responses under physiological and pathological conditions, this work highlights their potential to modulate a range of targets, including structural components (e.g., intercellular junctions), transmembrane proteins (e.g., channels and efflux pumps), intracellular regulators (e.g., transcription factors), and extracellular signaling mediators (e.g., cytokines and growth factors). Furthermore, it identified a nuanced, context-dependent function for histone and non-histone protein acetylation/deacetylation regarding BBB integrity, rather than a linear pattern, suggesting that non-selective modulation of HATs/HDACs may not be the most suitable therapeutic approach.

There has been a growing interest in pharmacological modulators targeting these mechanisms, particularly through HDAC inhibition. The FDA/EMA have already approved HDAC inhibitors for cancer-related treatments, and more recently for a neuromuscular disorder. Others are being investigated for additional therapeutic uses, offering a promising path for conditions involving a compromised BBB integrity. It is worth noting that this field is moving toward class or isozyme-specific HDAC inhibitors, since it was hypothesized that this might overcome the current challenges associated with pan-inhibitors, namely off-target effects, and toxicity. However, high sequence homology between catalytic sites, along with HDAC tendency to form functionally active multiprotein complexes necessary for their function, has been restricting the development of isozyme-selective inhibitors. It is also noteworthy that the use of physiologically relevant models of the BBB could be advantageous to overcome translational hurdles and broaden the understanding of its epigenetic regulation.

Ultimately, this work highlighted an evolving field with potential to transform current approaches to CNS disorders.

## Data Availability

No datasets were generated or analyzed during the current study.

## Abbreviations

ABCB1: ATP binding cassette subfamily B member 1
Angpt2: Angiopoietin-2
AQP4: Aquaporin-4
Arg1: Arginase 1
AHR: Aryl hydrocarbon receptor
BEC: Brain endothelial cell
BBB: Blood-brain barrier
BRD4: Bromodomain-containing protein 4
cAMP: Cyclic adenosine monophosphate
CBF: Cerebral blood flow
CBP: CREB-binding protein
CCM: Cerebral cavernous malformation
CNS: Central nervous system
CYP: Cytochrome P450
EAE: Experimental autoimmune encephalomyelitis
EMA: European Medicines Agency
FDA: Food and Drug Administration
FITC: Fluorescein isothiocyanate
GABA: Gamma-aminobutyric acid
GCN5: General control of amino acid synthesis 5
GDNF: Glial cell line-derived neurotrophic factor
GFAP: Fibrillary acidic protein
H3K14: Histone H3 at lysine 14
H3K27: Histone H3 at lysine 27
H3K27ac: Acetylation of lysine 27 on histone H3
H3K9: Histone H3 at lysine 9
H3K9ac: Acetylation of lysine 9 on histone H3
H4K12: Histone H4 at lysine 12
H4K5: Histone H4 at lysine 5
HAT: Histone acetyltransferase
HBO1: Histone acetyltransferase binding to ORC1
hCMEC: Human cerebral microvascular endothelial cells
HDACs: Histone deacetylases
I/R: Ischemia/reperfusion
IL: Interleukin
iNOS: Inducible nitric oxide synthase
iPSC: Induced pluripotent stem cell
Keap1: Kelch-like ECH-associated protein 1
Kp,uu: unbound brain-to-plasma ratio
lncRNA: Long non-coding RNA
LPS: Lipopolysaccharide
LSD1: Lysine specific demethylase 1
miRNAs: microRNAs
MLC2: Myosin II regulatory light chain
MMP: Matrix metalloproteinase
mRNA: Messenger RNA
MYST1/KAT6A: Member of MYST family/lysine acetyltransferase 6 A
NAD: Nicotinamide adenine dinucleotide
ncRNA: Non-coding RNA
NF-κB: Nuclear factor kappa B
NG2: Nerve/glial antigen 2
NgBR: Nogo-B receptor
NLRP3: NOD-,LRR- and pyrin domain-containing protein 3
Nrf2: Nuclear factor erythroid-2 related factor 2
OGD/R: Oxygen-glucose deprivation and reoxygenation
p300: E1A, associated protein p300
pCREB: Phosphorylated cAMP response element-binding protein
PET: Positron emission tomography
PGC: Peroxisome proliferator-activated receptor-γ coactivator
piRNA: Piwi-interacting RNA
PPARγ: Peroxisome proliferator-activated receptor gamma
PRC2: Polycomb repressive complex 2
PRMTs: Protein arginine methyltransferases
RCOR1: REST corepressor 1
RhoA: Ras homolog gene family member A
ROCK: Rho-associated kinase
RT-qPCR: Reverse transcription quantitative polymerase chain reaction
SIR: Silent information regulator
siRNA: Small interfering RNA
SIRT: Sirtuin
sncRNA: Short non-coding RNA
snoRNA: Small nucleolar RNA
Sox2: Sex-determining region y-box 2
TEER: Transendothelial electrical resistance
TET: Ten-eleven translocation methylcytosine dioxygenase
TGF-β: Transforming growth factor-β
TLR4: Toll-like receptor 4
TNF-α: Tumor necrosis factor-α
tRNAs: Transfer RNAs
VE: Vascular endothelial
VEGF: Vascular endothelial growth factor
Zn: Zinc
ZO-1: Zonula occludens-1
